# DNA Binding in High Salt: Analysing the Salt Dependence of Replication Protein A3 from the Halophile *Haloferax volcanii*

**DOI:** 10.1155/2012/719092

**Published:** 2012-09-03

**Authors:** Jody A. Winter, Bushra Patoli, Karen A. Bunting

**Affiliations:** Centre for Genetics and Genomics, University of Nottingham, Queen's Medical Centre, Nottingham NG7 2UH, UK

## Abstract

Halophilic archaea maintain intracellular salt concentrations close to saturation to survive in high-salt environments and their cellular processes have adapted to function under these conditions. Little is known regarding halophilic adaptation of the DNA processing machinery, particularly intriguing since protein-DNA interactions are classically salt sensitive. To investigate such adaptation, we characterised the DNA-binding capabilities of recombinant RPA3 from *Haloferax volcanii* (HvRPA3). Under physiological salt conditions (3 M KCl), HvRPA3 is monomeric, binding 18 nucleotide ssDNA with nanomolar affinity, demonstrating that RPAs containing the single OB-fold/zinc finger architecture bind with broadly comparable affinity to two OB-fold/zinc finger RPAs. Reducing the salt concentration to 1 M KCl induces dimerisation of the protein, which retains its ability to bind DNA. On circular ssDNA, two concentration-dependent binding modes are observed. Conventionally, increased salt concentration adversely affects DNA binding but HvRPA3 does not bind DNA in 0.2 M KCl, although multimerisation may occlude the binding site. The single N-terminal OB-fold is competent to bind DNA in the absence of the C-terminal zinc finger, albeit with reduced affinity. This study represents the first quantitative characterisation of DNA binding in a halophilic protein in extreme salt concentrations.

## 1. Introduction

During the normal cellular processes of replication, recombination and repair DNA transiently occurs in a single-stranded (ss) form, which is inherently more vulnerable to damage than double-stranded DNA (dsDNA). Across all domains of life, ssDNA-binding proteins (SSBs) play a crucial role in the protection of this exposed form, binding ssDNA with high affinity, consequently protecting against inappropriate secondary structure and annealing events and damage or modification of exposed bases [1]. In addition to this protective function, SSBs also have a regulatory role, in the organisation of sequential events in these complicated processes via protein-protein interactions [1].

Despite the central role SSBs play in DNA replication and recombination events, substantial variation has been observed in the SSBs across the three domains of life, with a degree of structural conservation in the oligo-nucleotide/-saccharide binding OB-fold [2]. The *E. coli *SSB forms a homotetramer, each monomer containing one OB-fold and an extended C-terminal domain involved in protein-protein interactions [3, 4]. The eukaryotic and archaeal SSBs are known as replication protein A (RPA) with eukaryotic RPAs typically consisting of a heterotrimer [1]. Zinc finger motifs are a common feature in both archaeal and eukaryotic RPAs.

Wider variation in subunit composition is seen in the archaea [5, 6]. The crenarchaeal *Sulfolobus solfataricus* (SsoSSB) protein consists of a monomer containing a single OB-fold. The domain organisation is considered to be more typical of bacterial SSBs, although the OB-fold is structurally more similar to that seen in eukaryotic RPAs [7]. Far greater diversity is observed in the euryarchaea. The *Pyrococcus furiosus* RPA is a stable heterotrimer [8], whereas the *Methanococcus jannaschii *RPA appears to be monomeric in form [9]. Unusually, *Methanosarcina acetivorans *(Mac) possesses three RPAs (RPA1, RPA2, and RPA3). Unlike eukaryotes, each of these can act as a separate SSB, forming homodimers, and additionally homotetramers in the case of RPA1 [10].

Investigating the differences and similarities in these essential proteins with a common core motif but diverse organisation is crucial to our understanding of both the evolution and mechanisms of DNA information processing. There is substantial further interest in how archaea, many existing in extreme environments, have adapted their fundamental processes to function under these conditions, such as in the euryarchaeal halophiles, emerging as genetically tractable model organisms [11]. These halophiles require high-salt concentrations for growth and maintain intracellular salt concentrations of K^+^ and Cl^−^ ions near saturation to combat the osmotic stress their extracellular environment places them under [12]. 

Various strategies for protein adaptation to a halophilic lifestyle have been noted, including an increase in acidic residues on the protein surface, an ordered solvent network, counter binding of ions, and an increase in intersubunit ion pairs but these are not universal [13–16]. Given that protein-DNA interactions are largely electrostatic in nature and, as such, are known to be sensitive to high-salt concentration, it is of significant interest to discover the strategies employed by halophiles to overcome these difficulties.

The affinity of the TATA-binding protein of the halophilic thermophile *Pyrococcus woesei* for DNA has been shown to increase with increased salt concentration over a range 0.8 to 1.2 M [17]. This phenomenon appears to be dependent on cation binding between the negative charges on the protein and DNA surface [17]. Further mutational analysis demonstrated that mutation of only three residues was sufficient to reverse the halophilic nature of binding, presumably due to a reduction in cation binding in the absence of the mutated glutamate side chains [18, 19]. At 0.8 M, the *P. woesei* intracellular salt concentration is more moderate than *Haloferax volcanii *at 2.1–4 M KCl [17, 20]. Characterisation of a DNA ligase from *H. volcanii *found maximal DNA strand-joining activity in 3.2 M KCl, with activity abrogated in the absence of salt [21].

Our recent crystal structure of the *H. volcanii* PCNA also suggested that cation binding compensates for the reduction in positively charged side chains observed in the central channel of this sliding clamp, reducing charge repulsion with the DNA backbone [16]. 

Proteins isolated from extremophiles are relevant in biotechnology where proteins naturally adapted to function under extremes, such as heat, have revolutionised many procedures [22]. Halophilic proteins would clearly be suited to procedures requiring high salt levels but have also been suggested to be relevant to environments that exclude water, such as organic solvents [23, 24]. 

Analysis of the DNA-binding capabilities of halophilic proteins is challenging since DNA-protein binding is classically salt sensitive and protocols typically aim to limit salts. Additional complications arise where salt adversely affects equipment, particularly overheating during electrophoresis. 

We have overexpressed and purified recombinant RPA3 from *H. volcanii* (HvRPA3), the smallest of the three *H. volcanii* RPAs, to adapt existing methodology to high salt conditions and explore the DNA-binding capabilities of the protein under a range of salt concentrations.

## 2. Materials and Methods

### 2.1. Cloning, Expression, and Purification of HvRPA3

Full-length HvRPA3 was amplified from *H. volcanii* genomic DNA (wild-type DS2 [12]) and cloned into the NdeI and KpnI sites of pETDuet1 (Novagen). The N-terminal domain (NTD) residues 1–163 and C-terminal domain (CTD) residues 164–311 regions were amplified from the full-length construct and cloned into the NdeI/XhoI sites of pETDuet1. All constructs contained an N-terminal 6xHisTag introduced via PCR. Overexpression was performed using *E. coli* B834 (DE3) cells grown in LB media containing 100 *μ*g/mL ampicillin. Full-length and CTD-expressing strains were grown to an OD_600_ of 0.6–0.8 and induced with 1 mM IPTG. Cells were further incubated at 37°C for 3-4 hours and harvested by centrifugation. To eliminate the breakdown product observed when the NTD was overexpressed under these conditions, cells were grown to an OD_600_ of 1.0–1.2 and induced with 1 mM IPTG at 25°C for 1 hour. The cell pellets were resuspended in buffer A (50 mM HEPES pH 7.0, 1.0 M NaCl, 10 mM imidazole) with an EDTA-free protease inhibitor cocktail tablet (Roche) and lysed via sonication, followed by clarification by centrifugation. Ammonium sulphate fractionation was performed and the 20–60% pellet (full length) and 0–60% pellets (NTD and CTD) were resuspended in buffer A and applied to Talon metal affinity resin (Clontech) in batch equilibrated in buffer A and incubated with rolling at room temperature for 30 minutes. The resin was washed with 30 column volumes of buffer A and eluted in 13 mL buffer B (buffer A supplemented to 300 mM imidazole). The resin was incubated in buffer B for 10 minutes to maximise elution. The eluted protein was applied to a 26/60 Superdex 200 column (GE Healthcare) equilibrated in 50 mM Hepes pH 7.0 and 1.0 M NaCl and run at 2 mL/min. Fractions were pooled and concentrated using a Vivapore 10/20 7500 Da cutoff (Vivascience) and proteins stored at 4°C. Protein concentration was assessed by UV absorption at 280 nm, correcting for individual extinction coefficients, and corroborated by scanning densitometry of the proteins compared to known standards via SDS-PAGE gels stained with SimplyBlue Safestain (Invitrogen).

### 2.2. Analytical Size Exclusion Chromatography

Analytical size exclusion chromatography (SEC) was performed on a 10/300 Superdex 200 column (GE Healthcare) equilibrated in buffer containing 50 mM HEPES pH 7.0 and either 0.2, 1.0, or 3.0 M KCl. 250 *μ*g of concentrated protein was diluted into the respective buffer and incubated for an hour prior to loading. Where relevant, equimolar amounts (monomer equivalent) of ssDNA (18mer) oligonucleotide (sequence 5′-GCGTGTGTGGTGGTGTGC-3′, (MWG Biotech)) were added prior to dilution in the respective buffer. Flow rate was maintained at 0.5 mL/min. Protein in fractions was monitored using SDS-PAGE analysis and the presence of ssDNA was monitored using the Qubit ssDNA kit (Invitrogen). Calibration was performed using gel filtration standards (BioRad) in each buffer to account for differential salt effects. Elution of the nonhalophilic molecular weight standards (Mr = 670 to 17 × 10^3^) was identical in all buffer conditions, with some variation observed in the 1.35 × 10^3^ standard.

### 2.3. Intrinsic Fluorescence Spectroscopy

Spectra were obtained using a Cary Eclipse fluorescence spectrophotometer with a 96-well plate reader attachment. An excitation wavelength of 280 nm was used and emission spectra were collected from 300 to 400 nm. Excitation and emission slits were both set at 5 nm. Full length HvRPA3 or HvRPA3-NTD were diluted in triplicate into buffer containing 50 mM HEPES pH 7.0 and either 0.2, 1.0, or 3.0 M KCl to give a final protein concentration of 70 *μ*M. Samples were incubated at room temperature for 18 hours prior to analysis. All spectra were corrected by subtraction of wells containing the storage buffer diluted into the sample buffers.

### 2.4. Fluorescence Anisotropy

The specified amounts of protein were titrated into reactions containing 50 mM HEPES pH 7.0, 10% glycerol, 0.03% BSA, 20 nM Cy5-labelled oligonucleotide sequence as above (MWG Biotech), and either 0.2, 1, or 3 M KCl. Reactions were incubated at room temperature for 10 minutes prior to reading. Anisotropy was measured in an EnVision 2102 Multilabel Reader (Perkin Elmer) plate reader with 620 nm excitation and 688 nm emission. To fit binding curves using GraphPad Prism v 5.0, a minimum of five datasets were averaged and normalised to account for viscosity differences between salt concentrations. Data were fit with a Hill binding model where *Y* = *B*_max⁡_∗*X* ^*h*^/(*K*_*d*_^*h*^ + *X* ^*h*^).   *B*_max⁡_ is the maximum specific binding, *K*_*d*_ is the concentration required for half-maximum binding, and *h* is the Hill slope. For the NTD in 1 M KCl a two site model was used (*Y* = (*B*_maxHi_∗*X*)/(*K*_*d*Hi_ + *X*) + (*B*_maxLo_∗*X*)/(*K*_*d*Lo_ + *X*)).

### 2.5. Agarose Gel Retardation

500 ng of PhiX174 ssDNA (New England Biolabs) was incubated with the indicated amounts of HvRPA3 in 20 mM Tris, 15 mM MgCl_2_, 2 mM DTT, 50 *μ*g/mL BSA, 6% glycerol, and 1 M KCl for 10 minutes at 37°C prior to loading on a 0.6% agarose gel. Following electrophoresis in 1x TBE buffer, DNA was visualised via ethidium bromide staining under UV illumination.

### 2.6. Zinc Detection by Inductively Coupled Plasma Mass Spectroscopy

Concentration of trace elements (Mn, Fe, Cu, Zn, Se, Mo, and Pb) in purified protein were determined by inductively coupled plasma mass spectrometry (ICPMS; Thermo-Fisher model XSeriesII) operating in “collision cell” mode (7% H_2_ in He) with “kinetic energy discrimination” (CCT-KED) to minimise polyatomic interferences. Internal standards (193Ir, 103Rh, 71Ga, and 45Sc) were added directly to both calibration standards and samples as constituents in a diluent solution (1/20) containing 1% Trace Analysis Grade HNO_3_ (Fisher Scientific), 2% methanol, and 0.1% nonionic surfactant “Triton-X.” 250 *μ*g of protein was diluted prior to addition of the diluent solution to a final NaCl concentration of 0.1 M. Samples were analysed in triplicate.

### 2.7. Homology Modelling

The region comprising the principal secondary structural elements of the OB-fold domain of HvRPA3 (76–170) was submitted to the *i*-Tasser server [25]. *i*-Tasser utilises a robust metathreading approach to identify potential templates. *i*-Tasser identified three templates: 2KEN/2KBN (OB-fold of MM0293 from *Methanosarcina mazei*) and 1O7I (*S. solfataricus* SSB), yielding a single model. Analysis using MolProbity produced a geometry score on the 47th centile and a clash score on the 99th centile [26]. The model is strongly supportive of the predicted OB-fold of this region. Comparison with the first OB-fold of human RPA70, not employed as a template, (1JMC [27]), using secondary structure matching yields an rmsd of 1.92 Å over 87 C*α* residues.

## 3. Results

**Figure 1:**
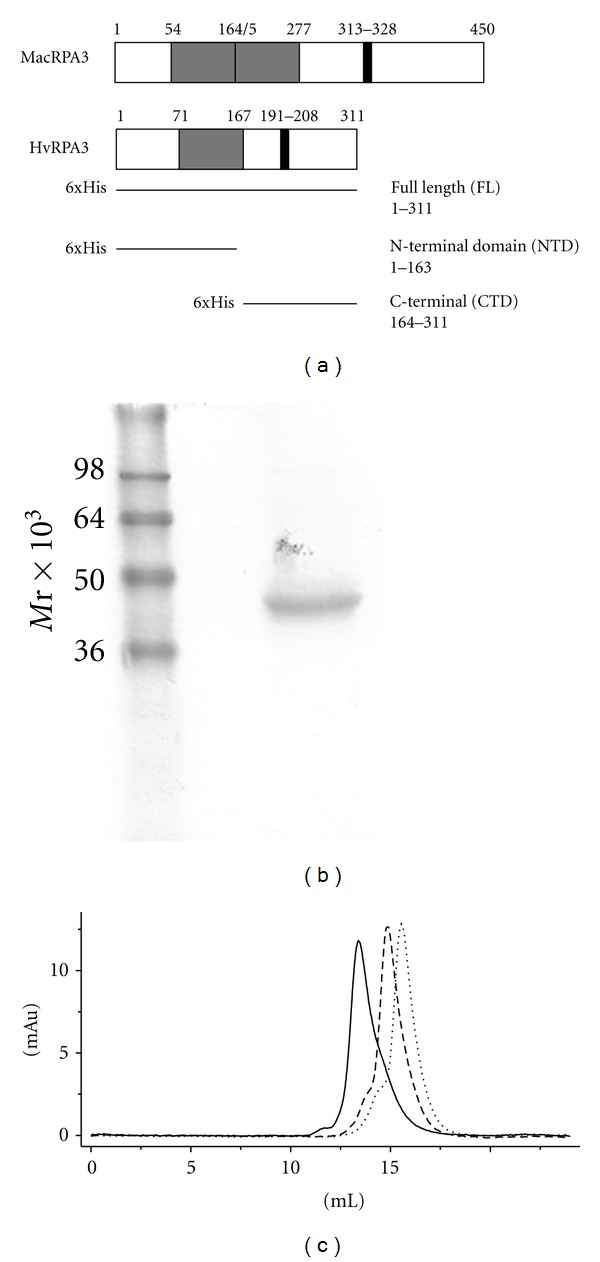
(a) Schematic representation of the arrangement of OB-folds (light grey) and CCCH zinc fingers (dark grey) and respective amino acid positions in HvRPA3 and MacRPA3 [10]. Constructs used in this study are indicated. (b) SDS-PAGE analysis of 2.5 *μ*g purified full length HvRPA3. The 6xHis-tagged construct has a predicted MW of 35.4 × 10^3^. (c) Size exclusion profiles of HvRPA3 in 0.2 M (solid line), 1 M (dashed line), and 3 M (dotted line) KCl. The *x* axis shows elution volume (mLs) and the *y* axis absorbance at 280 nm.

The RPA protein family has been widely studied due to its essential role in protecting ssDNA during DNA replication and repair and as a model system to understand adaptations of the widely found OB-fold. In particular, wide variation is seen in the euryarchaeota, in the composition and arrangement of RPA proteins. *H. volcanii* contains three putative RPA proteins, RPA1, RPA2, and RPA3, related to the three MacRPA proteins that were shown to function as separate SSBs [10, 28]. We selected HvRPA3 as a suitable target to explore DNA binding in halophiles since HvRPA3 is the smallest of the three *H. volcanii* proteins and contains a single OB-fold and zinc finger, with this arrangement being previously uncharacterised (Figure 1(a)). Sequence alignment of both RPA3 proteins (See Figure  1 in Supplementary Material available online at doi:10.115/2012/719092) suggests conservation at the N-terminus, with Robbins and others finding that residues 1–57 mediate dimerisation, and in the C-terminal region with both proteins possessing a zinc finger motif [6]. HvRPA3 contains a small insertion between the first two cysteine residues (Cx_4_Cx_8_Cx_2_H) relative to MacRPA3 (Cx_2_Cx_8_Cx_2_H). Motif analysis by InterProScan (http://www.ebi.ac.uk/Tools/pfa/iprscan/) predicts a single OB-fold for HvRPA3 (71–167) principally aligning with the second of the two MacRPA3 OB folds (54–164 and 165–277). 

Only the OB-fold domain of HvRPA3 has sufficient homology to solved structures to permit further investigation of the halophilic adaptation of this fold by homology modelling. The *i*-Tasser server identified three template structures, two NMR structures from the related *M. mazei* OB-folds and SsoSSB. Aligning the model to the first of the OB-fold domains (DNA-binding domain 1-DBD1) of the human RPA70 structure solved in complex with 8mer ssDNA suggests that the core fold of a closed *β*-barrel is conserved in HvRPA3 [27]. Deviations are observed in the connecting loop regions, as has been observed previously, although such regions are inevitably modelled with a reduced degree of confidence [1]. Structural analysis of SsoSSB implicated residues W56 and F79 as central to DNA binding; the aromatic nature of these is conserved in HvRPA3, possessing F120 and Y144, respectively [7]. F120 is equivalent to F238 which is involved in base stacking in human RPA70 [27].

A reduction in surface-exposed lysine residues is a common feature of halophilic adaptation and is particularly relevant in DNA-binding proteins where positive residues play a central role in the maintenance of electrostatic interactions with the negatively charged DNA backbone. Overall, HvRPA3, and MacRPA3 have an acidic nature, with theoretical pIs of 4.20 and 5.31, respectively. Focussing on the known DNA-binding motif, the OB-fold, comparing HvRPA3, MacRPA3 and known structures of *M. mazei*, human RPA70, and SsoSSB shows that both HvRPA3 and MacRPA3 have a pronounced reduction in lysine 4.2% and 3.9% versus 6.8–8.4% (Supplementary Figure  2). HvRPA3 compensates with the typical halophilic increase in aspartate, accounting for the reduced pI for this domain in HvRPA3 (4.03) as opposed to MacRPA3 at 6.55. These differences are reflected in the overall charge distribution shown in Supplementary Figure  3, with a portion of bound DNA shown for orientation on human RPA70 DBD1.

We have previously suggested that key positively charged residues involved in DNA interaction are retained, despite the increase in acidic character of halophilic proteins [16]. DNA binding by an OB-fold is well characterised in the cocrystal structure of human RPA70. Alignment with the RPA70 DBD1 shows that residues known to be involved in DNA binding (R210 and R234) are conserved as positive residues in HvRPA3 and SsoSSB. K263 (RPA70) has a potential structurally equivalent lysine in HvRPA3. Intriguingly, two lysine residues on the face distal to the DNA-binding cleft are retained, equivalent to human RPA70 R202 and K253, with R202 also conserved in SsoSSB, hinting at a conserved function, potentially in alternate binding modes or in protein-protein interactions.

### 3.1. Overexpression and Purification of HvRPA3

Pure protein was obtained using the described procedures, of greater than 95% purity as judged by SDS-PAGE (Figure 1(b)). Migration in a reducing gel is clearly retarded, the predicted molecular weight (MW) of the tagged protein being 35.4 × 10^3^. This phenomenon is frequently observed in halophilic proteins, since the increase in negatively-charged residues impedes SDS binding to the protein and therefore hinders migration [29].

### 3.2. Reducing Salt Concentration Promotes Multimerisation of HvRPA3

During purification, HvRPA3 consistently eluted from a 26/60 Superdex 200 column at a position equivalent to a dimer. Robbins and others observed that MacRPA3 appeared to be dimeric in form [10]. To explore this further with HvRPA3, analytical SEC profiles were compared under differing salt conditions (Figure 1(c)). KCl concentrations of 0.2, 1, 2, and 3 M were utilised since KCl concentration is more physiologically relevant to intracellular halophilic proteins than NaCl [20]. Well-defined peaks were observed under all buffer conditions.

The elution volume in 0.2 M KCl yields a predicted MW of approximately 135 × 10^3^, consistent with HvRPA3 forming a tetramer at this salt concentration. In 1 M KCl, the peak elutes consistently with a protein of 60 × 10^3^, indicating a dimer. In 3 M KCl, HvRPA3 was monomeric, eluting with a predicted MW of 30 × 10^3^. Identical results were seen at equivalent concentrations of NaCl (data not shown).

### 3.3. Increased KCl Concentrations Promote ssDNA Binding

**Figure 2:**
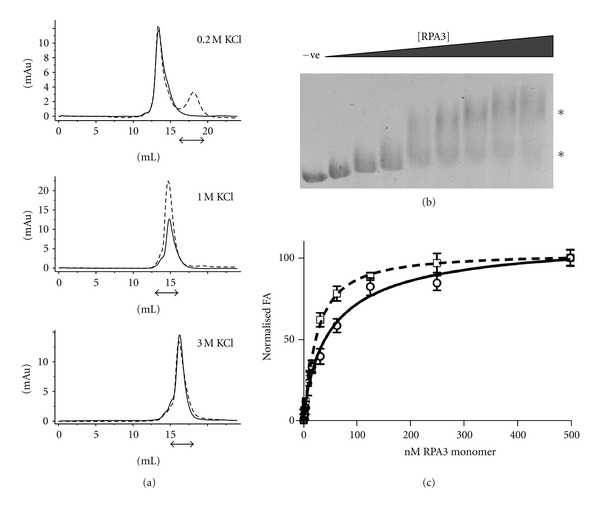
(a) Size exclusion profiles of HvRPA3 in the presence (dashed line) and absence (solid line) of equimolar (based on monomeric protein concentration) 18mer ssDNA in 0.2, 1, and 3 M KCl. The *x* axis shows elution volume (mls) and the *y* axis absorbance at 280 nm. Arrows indicate the elution position of ssDNA. (b) Agarose gel retardation assay of HvRPA3 titrated into reactions containing PhiX174 ssDNA. −ve indicates negative control containing no HvRPA3. Increasing quantities of HvRPA3 were used −11, 22, 33, 55, 75, 98, 120, and 125 *μ*g. Asterisks indicate the concentration-dependent, differentially migrating forms of complex. (c) Titration of the indicated concentrations of HvRPA3 (calculated for monomer due to the potential independence of binding sites in the dimeric form) plotted against normalised FA of the Cy5-labelled 18mer in 1 M (solid line) and 3 M (dashed line) KCl. Data were fitted to a Hill binding model. Produced using GraphPad Prism.

To compare binding of HvRPA3 to ssDNA under these KCl concentrations, SEC was repeated with equimolar (monomer equivalent) concentrations of an 18mer ssDNA (Figure 2(a)). In 0.2 M KCl, no binding was observed, with the protein peak and DNA peak each eluting separately. In contrast in 1 M KCl, a single peak of coeluting protein/ssDNA was observed, suggesting that the protein remains dimeric upon DNA binding. A shift in the elution volume and an increase in absorbance were observed upon DNA binding, from 12.7 to 22.5 mAu. In 3 M KCl, again the ssDNA oligo coelutes with the protein peak and a small shift in peak position is observed. The protein appears to remain monomeric. In this instance, no increase is observed in the absorbance of the complex versus the protein alone. No change in absorption was noted in oligonucleotide controls run under the varying salt conditions. Decreased UV absorption by intercalated bases versus those unstacked in single-stranded DNA is a known phenomenon and the results here are likely to indicate differences in the exposure of bases between the monomeric and dimeric form [30]. 

### 3.4. HvRPA3 Shows Two Modes of DNA Binding

Electrophoretic mobility shift assays (EMSA) are commonly employed to characterize DNA binding. Adaptation of this technique to high salt conditions is challenging, since increasing salt level leads to overheating of the equipment. However, with optimisation, band shifts were observed with agarose gels run in 1× TBE buffer, following incubation of increasing quantities of HvRPA3 with PhiX174 ssDNA in 1 M KCl (Figure 2(b)). Protein concentrations sufficient to induce shifts are in significant excess to those expected based on similar experiments with mesophilic RPAs. This presumably reflects the suboptimal conditions for halophilic binding during electrophoresis, since it is not possible to maintain appropriate KCl levels in the running buffer. However, the system does permit qualitative analysis of binding, in that binding of the circular ssDNA approaches saturation suggesting the ssDNA is fully occupied by HvRPA3 molecules and that two differentially migrating complexes are observed, one only at higher protein concentration. 

### 3.5. Fluorescence Anisotropy Permits Characterization under High-Salt Conditions

To provide quantitative analysis under conditions representative of physiological salt levels, fluorescence anisotropy (FA) was employed to study binding using an 18mer oligonucleotide labelled at the 5′ end with a Cy5 fluorophore, broadly as described for MacRPA3 [10]. Analysis in 0.2 M KCl, even with high concentrations of protein, did not show saturating binding, in agreement with the SEC data (data not shown). Saturation of binding was observed in 1 and 3 M KCl (Figure 2(c) and Table 1). Binding models that account for overlapping binding sites and the consequent occlusion of possible binding sites have been employed to analyse eukaryotic RPA DNA binding [31]. However, in this case, while the size of the binding site can be predicted by comparison of the HvRPA3 OB-fold model with human RPA70, the extent of occlusion, and hence the number of remaining available binding sites, is not known. Therefore, given the length of oligonucleotide utilised and to minimise assumptions employed in model fitting, data were fit to a Hill binding model as for MacRPA3, with good agreement between the resulting curves and anisotropy data [10, 32].

This analysis yields a dissociation constant of 24.1 ± 2.9 nM and a Hill coefficient of 1.2 ± 0.1 for HvRPA3 in 3 M KCl. A value greater than 1 is suggestive of cooperative binding. The values obtained for MacRPA3 are indicated in Table 1, for comparison [10]. The same model yields values in 1 M KCl of 53.9 ± 18.3 nM. A Hill coefficient of less than 1 (0.9 ± 0.1) suggests that binding of one molecule makes a second molecule binding less energetically favourable and, for the purposes of this analysis, the individual subunits within the dimer were assumed to be independent units.

### 3.6. The DNA Binding Site Appears to Be Occluded under Low-Salt Conditions

**Figure 3:**
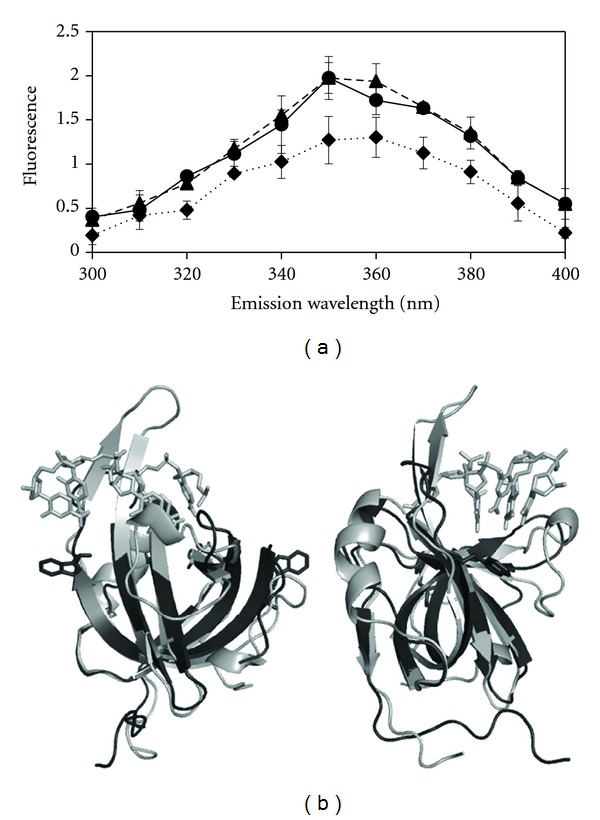
(a) Intrinsic fluorescence spectra for HvRPA3 incubated in 0.2 (dotted line), 1 M (dashed line), and 3 M (solid line) KCl. The *x* axis shows the wavelength of the emission spectra (nm) and the *y* axis the observed fluorescence. (b) Homology modelling of the HvRPA3 OB-fold (76–170). The HvRPA3 model (dark grey) and human RPA70-ssDNA (1JMC) complex (light grey) are shown in cartoon representation in two orthogonal views [27]. DNA is shown in stick form. The two tryptophan residues in HvRPA3 are shown in stick representation. 1JMC contains two OB-folds and a 8mer ssDNA, for clarity only the first OB-fold and the proximal 4 nucleotides are shown. Produced using PyMol [34].

Since DNA binding is abrogated in 0.2 M KCl and HvRPA3 appears to form tetramers under this condition, we wished to assess the accessibility of the binding cleft (Figures 3(a) and 3(b)). DNA binding in SSBs has been assessed by changes in the fluorescence emission spectra of tryptophan residues, with quenching occurring upon DNA binding [7, 33]. Inspection of the HvRPA3 OB-fold model when aligned with the human RPA70/DNA complex suggests the two tryptophan residues in HvRPA3 are proximal to bound DNA, although they are not located within the binding cleft (Figure 3(b)) [27].

The emission spectra for buffer conditions containing 1 and 3 M KCl were virtually identical (Figure 3(a)). A marked reduction in fluorescence intensity was observed in 0.2 M KCl, although the emission wavelength maxima remain similar under all three conditions and are reminiscent of the quenching observed during DNA binding in RPA proteins [33]. Previous RPA studies have shown that variation in monovalent ion concentration up to 3 M does not affect the emission spectra [31].

 These data, taken in conjunction with the SEC data, suggest that these tryptophan residues are less solvent exposed in the tetrameric form found in 0.2 M KCl, likely to result in at least partial occlusion of the DNA-binding site, since they are positioned flanking this site. Despite dimerisation, in 1 M KCl, the emission profile of the two tryptophan residues is identical to that of the monomeric 3 M KCl form, consistent with both forms being proficient for DNA binding, although it must be emphasised that the tryptophan residues are not implicated in direct DNA binding, merely as markers flanking the DNA-binding cleft.

### 3.7. Reducing Salt Concentration Induces Multimerisation of the N- and C-Terminal Domains

**Figure 4:**
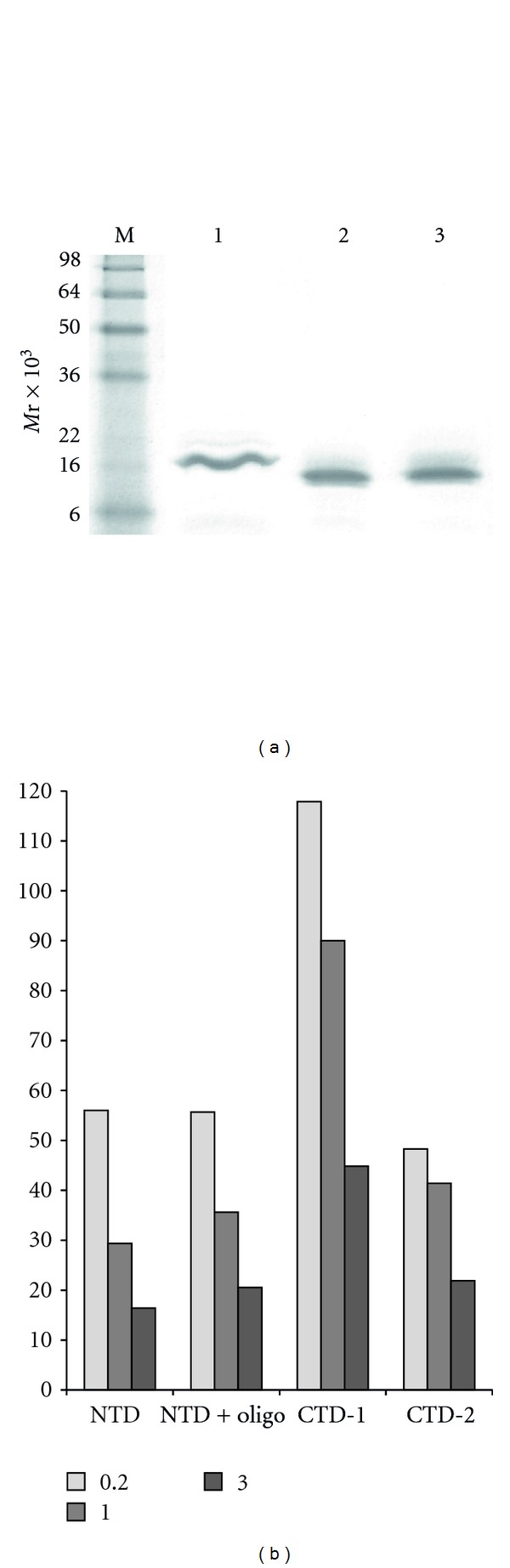
(a) SDS-PAGE analysis of 2.5 *μ*g purified HvRPA3-NTD, HvRPA3-CTD-1, and HvRPA3-CTD-2. The 6xHis-tagged constructs have predicted MWs of 18.9 × 10^3^ (HvRPA3-NTD) and 17.6 × 10^3^ (HvRPA3-CTD). (b) Predicted MWs (*y* axis, ×10^3^) from size exclusion chromatography analysis of HvRPA3-NTD, HvRPA3-CTD-1, and HvRPA3-CTD-2 in 0.2, 1, and 3 M KCl (light, mid, and dark grey, resp.). HvRPA3-NTD in complex with 18mer ssDNA is also shown.

To further dissect DNA binding and multimerisation, we over-expressed and purified N-terminal domain (NTD) and C-terminal domain (CTD) constructs (Figure 1(a)). The NTD contains the OB-fold domain, while the CTD contains the zinc finger motif. During purification, CTD produced two peaks of equivalent absorption during the final size exclusion step, with predicted MWs of 71.3 × 10^3^ and 34 × 10^3^, respectively, presumably representing tetramer and dimer of the 17.6 × 10^3^ protein. These two peaks were concentrated separately for analysis and were termed CTD-1 and CTD-2. CTD-1 and 2 appeared identical when analysed via SDS-PAGE, migrating as expected for a 17.6 × 10^3^ protein (Figure 4(a)). 

In 3 M KCl, NTD and CTD-2 appeared to elute from the size exclusion column at a position consistent with the monomer, with NTD eluting slightly later than predicted for a 18.9 × 10^3^ protein, suggestive of a more compact conformation under these conditions, since the protein appears intact when analysed by SDS-PAGE (Figures 4(a) and 4(b)). In 1 M KCl, the elution volumes for both NTD and CTD-2 suggest dimerisation. Both appeared to form trimers in 0.2 M KCl. In contrast, CTD-1 eluted in 3 M KCl between the volumes expected for a dimer and trimer. Higher order multimerisation of CTD-1 was observed in lower KCl concentrations, with pentamers in 1 M and heptamers in 0.2 M KCl.

### 3.8. The NTD of HvRPA3 Retains ssDNA-Binding Activity

NTD, CTD-1, and CTD-2 were analysed for ssDNA-binding propensity via SEC and FA as for the full-length protein. No evidence for DNA binding was obtained for either CTD-1 or CTD-2 protein under any condition (data not shown). 

**Figure 5:**
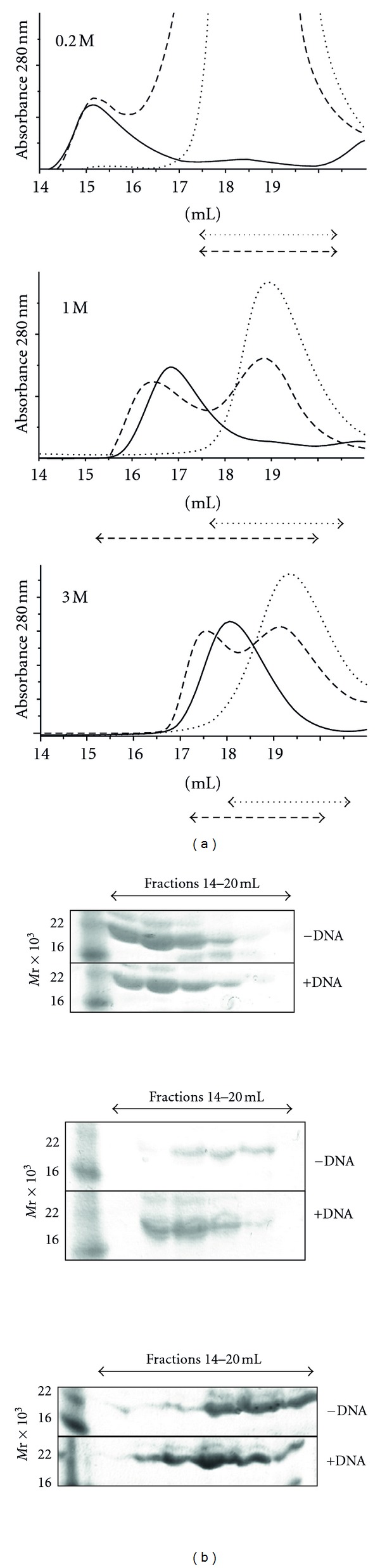
(a) Size exclusion profiles of HvRPA3-NTD in the presence (dashed line) and absence (solid line) of equimolar 18mer ssDNA in 0.2, 1, and 3 M KCl. The elution profile of the oligonucleotide alone is shown (dotted line). The *x* axis shows elution volume (mls) and the *y* axis absorbance at 280 nm. For clarity the peaks containing protein and protein/oligonucleotide have been scaled relative to the oligonucleotide alone. Peak heights (mAu) were 0.2 M 6.2/6.7, 1 M 7.4/40.5, and 3 M 10.1/59.3 (NTD alone/NTD+18mer ssDNA). Arrows indicate the presence of ssDNA in complex (dotted line) and alone (dashed line). (b) SDS-PAGE analysis of the indicated fractions in the presence and absence of oligonucleotide. Increased salt concentrations induce a degree of warping in the gels.

SEC analyses suggest that the NTD binds as a dimer in 1 M KCl and as a monomer in 3 M KCl, with no binding observed in 0.2 M KCl, as for the full-length protein (Figure 5(a)). Analysis by SDS-PAGE shows higher molecular weight bands that might indicate a residual dimeric form in 0.2 M KCl (Figure 5(b)). These are absent in 1 M KCl without ssDNA but appear after the addition of ssDNA. A band corresponding to a dimer is not immediately apparent in 3 M KCl fractions; however, the increased salt levels reduce band resolution and cause lane widening. The presence of unbound oligonucleotide may well reflect the reduction in binding affinity seen with the NTD alone, leading to dissociation of oligonucleotide from protein during the course of the experiment.

**Figure 6:**
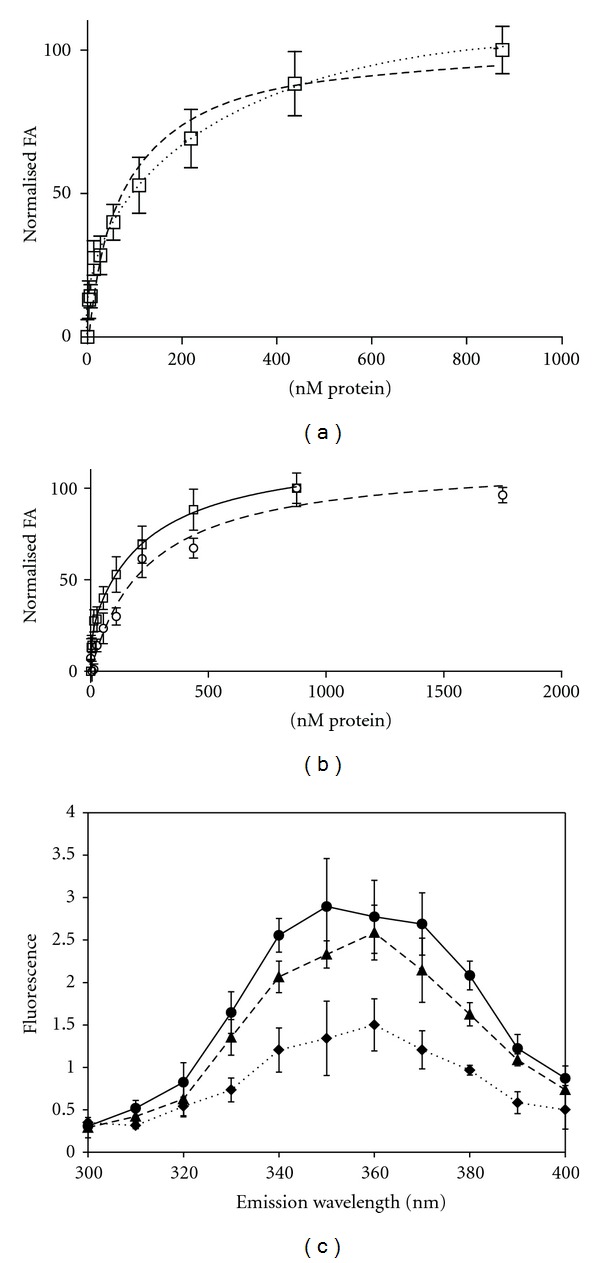
(a) Comparison showing the improved fit of a two-site binding model (dotted line) versus the Hill binding model employed for analysis (dashed line) for the HvRPA3-NTD dimer in 1 M KCl. The *x* axis displays the concentration of the dimer with the y axis indicating normalised FA. (b) Comparison of binding of dimeric HvRPA3-NTD in 1 M (solid line) fitted to the two-site model displayed in (a) and the monomeric form in 3 M (dashed line) KCl fitted to a Hill binding model. The *x* axis displays the concentration of the proteins with the y axis indicating normalised FA. (c) Intrinsic fluorescence spectra for HvRPA3-NTD incubated in 0.2 (dotted line), 1 M (dashed line), and 3 M (solid line) KCl. The *x* axis shows the wavelength of the emission spectra (nm) and the *y* axis the observed fluorescence.

Higher concentrations of NTD (1750 nM) were required to produce saturated binding curves by FA, compared to 500 nM for the full-length protein (Figures 6(a) and 6(b) and Table 1). While, as for MacRPA3, a Hill binding model produced curves in good agreement with the FA data for the HvRPA3 constructs, this was not the case for the NTD in 1 M KCl. A two site binding model is a closer fit to the 1 M KCl data than a single site binding model (Figure 6(a)), giving dissociation constants of *K*_*d*Hi_4.1 ± 7.9 nM, *K*_*d*Low_  246.6 ± 195.5 nM, with the large errors indicating that the model does not entirely describe the binding behaviour. For the monomeric 3 M KCl data, the Hill binding model produced a curve in good agreement with the data, yielding a dissociation constant of 212.5 ± 67.6 nM and a Hill coefficient of 1.1 ± 0.2, showing a reduction in affinity by an order of magnitude and a similar degree of positive cooperativity to the full-length protein. Intrinsic fluorescence analysis of NTD shows reduced fluorescence in 0.2 M KCl, presumably reflecting the multimeric status of this domain under these conditions (Figure 6(c)). Unlike the full length protein, in 1 M KCl, a slight reduction in fluorescence is observed compared to 3 M KCl, suggestive of a partial occlusion of one or both tryptophans upon dimerisation. 

### 3.9. Zinc Binding

ICP-MS was utilised to assess the zinc content of the HvRPA3 constructs (Table 2). As expected, NTD does not coordinate zinc and CTD-1 contained no detectable zinc. Zinc levels of 0.60 and 0.53 mol of zinc/mol of protein (monomer concentration) in full length and CTD-2, respectively, suggest that CTD-2 is the true representative of the domain. CTD-1 clearly retains some degree of secondary structural organisation due to its ordered multimerisation, possibly reflecting disulphide formation due to free cysteine residues.

## 4. Discussion

Although general trends in halophilic adaptation have been identified, there are no universal determinants, presumably a reflection on the diversity of protein structure and function in the intracellular environment. Structural analysis has found that the majority of halophilic proteins studied to date are broadly conserved architecturally in comparison to their nonhalophilic counterparts [35]. The acidic nature of halophilic proteins is largely attributable to an increase in surface-exposed negatively charged residues and is believed to limit protein aggregation [36]. Homology modelling suggests that HvRPA3 is typical of halophilic proteins in terms of its architectural conservation and increased acidic nature (Figure 3(b) and Supplementary Figures  2 and 3).

Modelling suggests that HvRPA3 could bind DNA in a manner seen in other RPA proteins, given the retention of both intercalating aromatic residues and a number of positively charged residues involved in binding. Nonetheless, the binding cleft shows a marked reduction in electropositivity, and high resolution co-crystal structures are required to dissect in detail the role residues flanking these conserved residues play and particularly to identify surface ions that have been suggested by previous studies to be an important mechanism of halophilic adaption of DNA binding [16, 18, 19].

Analysis of the *P. woesei* TATA-box binding protein demonstrated a strong trend of increasing affinity for DNA binding with increasing salt concentration (0.8 to 1.2 M) [17]. However, the intracellular salt concentration of *H. volcanii* is significantly higher than *P. woesei,* and we wished to extend our analysis to levels more appropriate to *H. volcanii*, up to 3 M KCl. Several studies of halophilic DNA binding enzymes have demonstrated increasing enzymatic activity to such levels, strongly inferring DNA binding under these conditions [21]. Practical difficulties in adapting established protocols to quantify DNA binding in 3 M NaCl/KCl have inevitably limited detailed analysis of binding. Using a combination of SEC, FA, and agarose gel retardation, we have characterised the binding of HvRPA3.

Under physiological salt conditions, in 3 M KCl, HvRPA3 appears to bind ssDNA as a monomer under the equimolar conditions employed in SEC analysis (Figure 2(a)). In the saturating binding conditions used for FA, the estimated dissociation constant is in the nanomolar range and is only slightly reduced compared to MacRPA3 (Table 1). It is clear that HvRPA3 has adapted to function under these extreme salt conditions, with broadly comparable affinity to MacRPA3. Indeed, the slight reduction may reflect the fact that HvRPA3 possesses only a single OB-fold compared to the two identified in MacRPA3. Model fitting yields a Hill coefficient greater than one, indicating positive cooperativity of binding, supported by the binding curve in 3 M KCl, suggestive of cooperative rather than independent binding, as observed for MacRPA3 (Figure 2(c)). Positive cooperativity has previously been reported for the monomeric SSB T4 gene 32 protein and the authors ascribe this effect to distortions to the DNA and/or direct protein-protein interactions that make subsequent binding of further molecules more favourable [37].

Analysis of the NTD suggests that the OB-fold domain is sufficient for DNA-binding activity, albeit with reduced binding affinity of an order of magnitude (Table 1), more pronounced than that observed in MacRPA3 (21.1 ± 0.8 nM [6]). Presumably, the effect is more profound for HvRPA3 than MacRPA3 since HvRPA3 possesses only a single OB-fold. The equivalent MacRPA3 C-terminal deletion containing a single OB-fold bound more weakly (879.0 ± 624.0 nM), presumably because this protein is optimised for two OB-fold binding.

HvRPA3 forms a dimer in 1 M KCl, seen in both the full-length protein and the NTD and appear by SEC to bind DNA as a dimer (Figures 2(a) and 5(a)). ssDNA was preincubated in an equimolar ratio relative to the concentration of monomer. For full-length HvRPA3, given the lack of residual oligonucleotide peak, this suggests that the ratio of binding is 1 dimer: 2 oligonucleotides. The increased absorption of the protein, DNA complex under these conditions, when compared to the complex in 3 M KCl, suggests a variation in binding between the two protein forms. We interpret this to indicate that, in 1 M KCl, a portion of the oligonucleotide is mobile, hence the increase in UV absorption. A Hill coefficient of 0.9 suggests a degree of negative cooperativity under conditions where an excess of protein is present, suggesting that binding of a protein ligand makes the binding of a second ligand less energetically favourable. This is in contrast to the situation observed in MacRPA3 (a dimer) and probably reflects alterations in HvRPA3 as a consequence of halophilic adaptation. The OB-folds within the dimer may not be optimally oriented for sequential binding and that binding between the sites is potentially independent. This does not appear to be the case for the NTD in 1 M KCl. Fitting a two-site binding model produces a curve of better fit to the FA data (Figure 6(a)), although the large errors indicate that this model does not entirely describe the binding behaviour. The slight reduction in tryptophan fluorescence observed in the NTD under 1 M KCl conditions suggests that the tryptophan residues are less solvent exposed in the NTD dimer than the full length dimer. Taking both these factors into account, it is plausible that the NTD dimer could bind ssDNA in tandem array as found in the human RPA70-DNA co-crystal structure [27]. In both 1 and 3 M KCl, an increase in absorption is seen on complexation of NTD and ssDNA, suggesting that a proportion of the 18mer oligonucleotide is mobile. This is consistent with the predicted binding footprint of the OB-fold relative to an 18mer oligonucleotide; the structure of human RPA70 contains tandem OB-folds binding an 8mer ssDNA molecule.

To examine binding in a context more relevant to cellular conditions, the effect of HvRPA3 on circular ssDNA was assessed by EMSA (Figure 2(b)). Although it is difficult to quantify the exact salt concentration the complexes experience during electrophoresis, two forms of complex are clearly visible. The slower migrating form appears only at higher protein concentrations. FRET analysis of MacRPA3 clearly demonstrated that the protein possesses two DNA binding modes, which are concentration-dependent [6, 10]. At low concentrations, a wrapping mode predominates. In contrast, at a critical concentration, the protein arranges itself such that the ssDNA becomes stretched and the protein molecules are presumably arranged in tandem array along the length of ssDNA. This latter form would migrate more slowly in EMSA. The presence of this slower migrating form at higher protein concentration suggests that HvRPA3 shows similar behaviour to MacRPA3, despite the variation in multimeric state and number of OB-folds complicating extrapolation.

Robbins and others proposed that the variation in wrapping and stretching modes would present alternate regions of RPA for interaction with protein partners or DNA and could affect ssDNA conformation [10]. It seems likely that in the organisms that possess several independent SSBs, like *M. acetivorans* and *H. volcanii*, precise control of each protein and consequently their partners' proteins will be crucial for temporal and spatial regulation of DNA processing. Robbins and others demonstrated that the C-terminal zinc finger region was required for both binding modes in MacRPA3 and for positive cooperativity [6]. Little difference in cooperativity is seen between the full-length HvRPA3 (1.2 ± 0.1) and NTD (1.1 ± 0.2) and this may reflect differences between the monomeric HvRPA3 and dimeric MacRPA3. The C-terminal domain of HvRPA3 binds zinc with comparable occupancy to the full-length protein and is likely to play a role in regulation of ssDNA binding via redox, as suggested for MacRPA3 [38].

Multimerisation associated with decreasing salt concentration is a marked feature of the constructs analysed in this study (Figures 1(c) and 4(b)). The sharp elution profiles observed are consistent with defined multimerisation rather than aggregation associated with the partial unfolding observed in some halophilic proteins at lower salt concentrations [39]. A similar increase in multimerisation at low salt concentration has been observed with *H. volcanii* DNA polymerase X and RadA in our hands (data not shown). Presumably at lower salt levels, the decoration of the protein surface with ions observed structurally [14, 16] is less than optimal, resulting in exposure of charged residues. In the case of DNA-binding proteins, which are more likely to retain positively charged residues in patches, multimerisation may be driven by salt bridge formation between basic patches and the largely negative surfaces of the neighbouring protein, resulting in the defined peaks observed, rather than aggregation of partially unfolded proteins due to exposure of hydrophobic core residues. 

No evidence was found for ssDNA binding in 0.2 M KCl (Figures 2 and 5). Some *H. volcanii* proteins have been shown to bind DNA in low salt conditions [40], whereas other enzymes have no activity in the absence of salt, potentially due to abrogation of DNA binding [21]. This variation likely reflects the diversity of both adaptation and protein function in the cell. Lack of DNA binding and the observed reduction in intrinsic fluorescence in both the full-length and NTD proteins under 0.2 M KCl conditions is consistent with at least partial occlusion of the DNA-binding cleft due to this defined multimerisation, although it is likely that suboptimal surface ion decoration under these conditions contributes to the lack of binding. 

Such association under low salt conditions is likely distinct from the dimerisation effect seen in 1 M KCl in both the full-length and NTD proteins. Robbins and others [6] identified the residues N-terminal to the first OB-fold as central to dimerisation and alignment supports conservation of this region in the single OB-fold RPAs. HvRPA3 is dimeric in 1 M KCl and presumably associates in a similar manner to MacRPA3. 3 M KCl conditions are likely refractory to formation of this dimer interface. Although increases in ion pairs have been commonly noted as a form of halophilic adaptation to stabilise interfaces under high salt conditions, it is not a universal effect. In this instance, the monomeric form has adapted to function in high salt concentrations, reflecting the range of adaptations and diversity of protein function in the cell. To further understand the diversity and adaptation of these proteins, it would be of interest to characterise the DNA binding and multimerisation behaviour of other single OB-fold archaeal RPAs, such as that of *Archaeoglobus* from a thermophilic, rather than halophilic source (Supplementary Figure  1). Clustering of the halophilic RPA3 proteins and the equivalent *Archaeoglobus* protein is consistent with phylogenetic analysis of other DNA replication proteins, such as the MCM complex [41]. 

As has been noted, the archaea present a melting pot for differing arrangements of SSBs and RPAs and are an excellent model to study the evolution of such a widespread fold as the OB-fold [6]. The two OB-fold/zinc finger arrangement in the well-characterised MacRPA3 appears to be the most common. This study represents the first characterisation of a single OB-fold-containing-RPA coupled with a zinc finger. It is also the first quantitative study of DNA binding under such extreme salt conditions and represents a significant step forward in the understanding of halophilic adaptation of this most classically salt-sensitive interaction, including the applicability of standard assays to characterise DNA binding under extreme salt conditions. Work is under way to exploit this information in structural studies, to provide detailed characterisation of a DNA protein co-crystal and fully dissect halophilic adaptation for DNA binding.

## Supplementary Material

Supplementary Figure 1: Sequence alignment of HvRPA3 with archaeal homologues using Clustal v2.0.12 on default settings and Boxshade v3.21. Brown boxes indicate the positions of the two OB-folds in MacRPA3, blue arrow the position of the HvRPA3 OB-fold. Additional labelled elements indicate the zinc ligands (red arrows) and N-terminal dimerisation domain (blue box).
Supplementary Table 2: Percentage amino acid usage over the OB-fold domains. Blue indicates residues discussed in the main text.
Supplementary Figure 3: Electrostatic surface potential of the HvRPA3 OB-fold model compared to related structures scaled at -10 kBT/e (red) to +10 kBT/e (blue). Human RPA70 shows a single OB-fold for clarity, with a 4mer stretch of oligonucleotide in stick representation in yellow. Produced using APBS and PyMol.

## Figures and Tables

**Table 1:** Dissociation constants and Hill coefficients calculated from a Hill binding model for HvRPA3 constructs compared with dimeric MacRPA3 [10]. A two-site binding model was employed for the NTD analysis in 1 M KCl as described in Section 2. Protein concentration is for the monomer equivalent, with the exception of NTD in 1 M KCl, where dimeric concentration was employed in model fitting.

[KCl]	*K*_*d*_ (nM)	Hill coefficient
Full length 1 M	53.9 ± 18.3	0.9 ± 0.1
Full length 3 M	24.1 ± 2.9	1.2 ± 0.1
MacRPA3	5.92 ± 0.23	1.46 ± 0.08
NTD 1 M		
High	4.1 ± 7.9	
Low	246.6 ± 195.5	
NTD 3 M	212.5 ± 67.6	1.1 ± 0.2

**Table 2:** ICP-MS analysis of zinc binding. Values shown are averages of triplicate samples of 250 *μ*g of the indicated protein in 500 *μ*L volume.

Protein	Mean Zn (*μ*g/L)	*μ*M Zn	Zn/mol
Full length	557.8 ± 4.8	8.53	0.60
NTD	104.0 ± 5.2	1.59	0.06
CTD-1	45.1 ± 2.1	0.69	0.03
CTD-2	787.1 ± 16.1	12.03	0.53
