# Supplementary material for: DNA Binding in High Salt: Analysing the Salt Dependence of Replication Protein A3 from the Halophile Haloferax volcanii
Source: Archaea. 2012 Sep 3;2012:719092. doi: 10.1155/2012/719092 (PMC3438722; doi:10.1155/2012/719092)
Supplement: Supplementary file 1 — Supplementary Figure 1: Sequence alignment of HvRPA3 with archaeal homologues using Clustal v2.0.12 on default settings and Boxshade v3.21. Brown boxes indicate the positions of the two OB-folds in MacRPA3, blue arrow the position of the HvRPA3 OB-fold. Additional labelled elements indicate the zinc ligands (red arrows) and N-terminal dimerisation domain (blue box). Supplementary Table 2: Percentage amino acid usage over the OB-fold domains. Blue indicates residues discussed in the main text. Supplementary Figure 3: Electrostatic surface potential of the HvRPA3 OB-fold model compared to related structures scaled at -10 kBT/e (red) to +10 kBT/e (blue). Human RPA70 shows a single OB-fold for clarity, with a 4mer stretch of oligonucleotide in stick representation in yellow. Produced using APBS and PyMol. [file 719092.f1.pdf]

Supplementary Material.

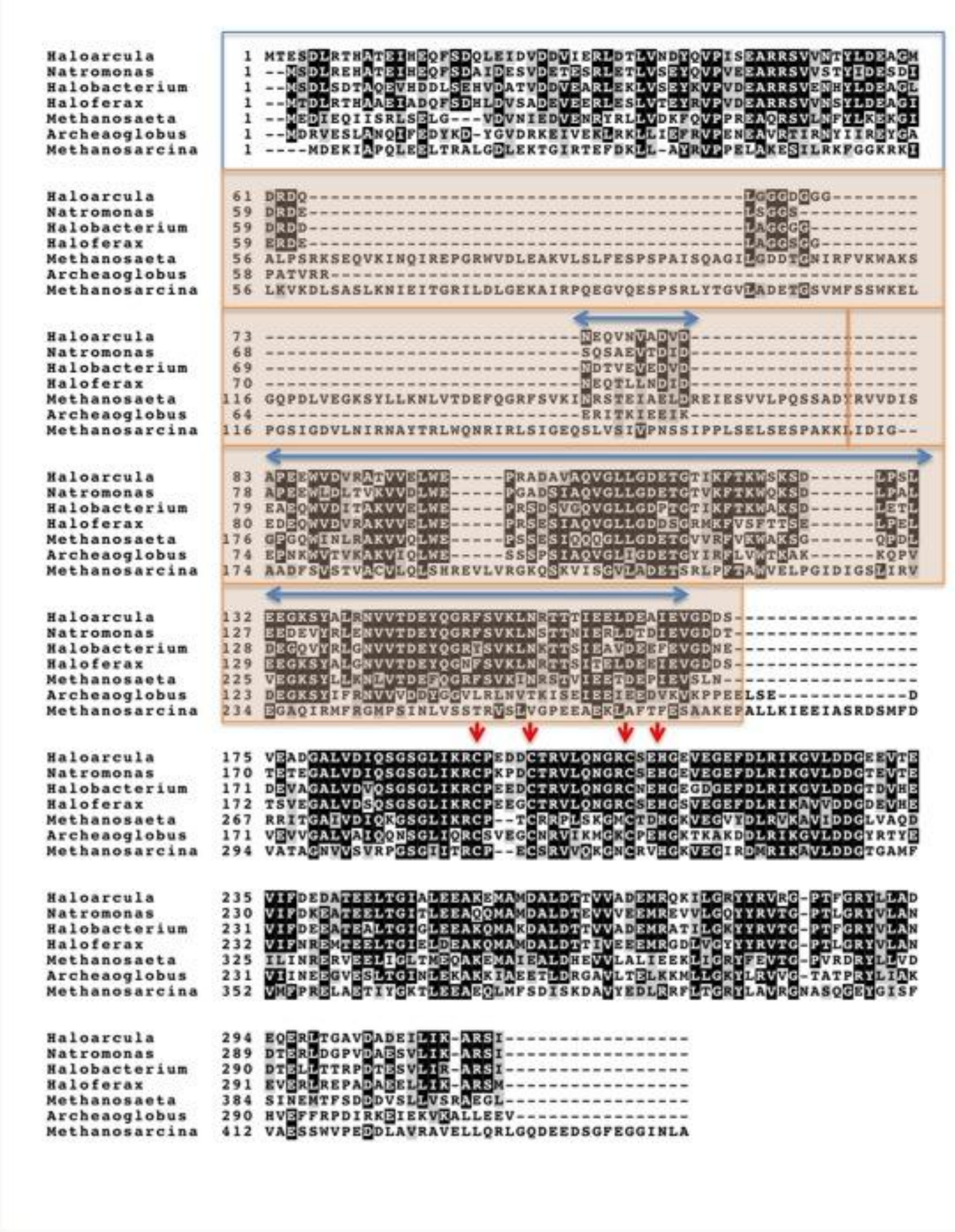

Supplementary Figure 1. Sequence alignment of HvRPA3 with archaeal homologues using Clustal v2.0.12 on default settings and Boxshade v3.21. Brown boxes indicate the positions of the two OB-folds in MacRPA3, blue arrow the position of the HvRPA3 OB-fold. Additional labelled elements indicate the zinc ligands (red arrows) and N-terminal dimerisation domain (blue box)

Supplementary Table 2. Percentage amino acid usage over the OB-fold domains. Blue indicates residues discussed in the main text.

	HvRPA3 (76-170)	MacRPA3 (180-258)	*M. mazei* (2KBN – chain A)	Human RPA70 (1JMC - 183-294)	SsoSSB (1O7I – chain A)
Ala	3.2	6.8	2.9	5.4	8.4
Arg	4.2	6.8	1.9	3.6	4.2
Asn	4.2	1	6.8	7.1	5.9
Asp	10.5	3.9	2.9	4.5	1.7
Cys	0	1	0	1.8	0
Gln	3.2	2.9	6.8	3.6	6.7
Glu	14.7	6.8	12.6	8	10.9
Gly	7.4	7.8	6.8	3.6	8.4
His	0	1	1	1.8	0.8
Ile	4.2	7.8	8.7	5.4	5.9
Leu	8.4	10.7	11.7	5.4	4.2
Lys	4.2	3.9	6.8	8.9	8.4
Met	1.1	1.9	1	1.8	2.5
Phe	3.2	2.9	1.9	6.2	1.7
Pro	2.1	4.9	2.9	3.6	4.2
Ser	8.4	13.6	6.8	8	6.7
Thr	6.3	3.9	3.9	8	8.4
Trp	2.1	1	2.9	1.8	1.7
Tyr	2.1	0	1.9	3.6	0
Val	10.5	11.7	9.7	8	9.2
pI	4.03	6.55	4.67	6.93	6.6
Asp+Glu	24	11	16	14	15
Arg+Lys	8	11	9	14	15

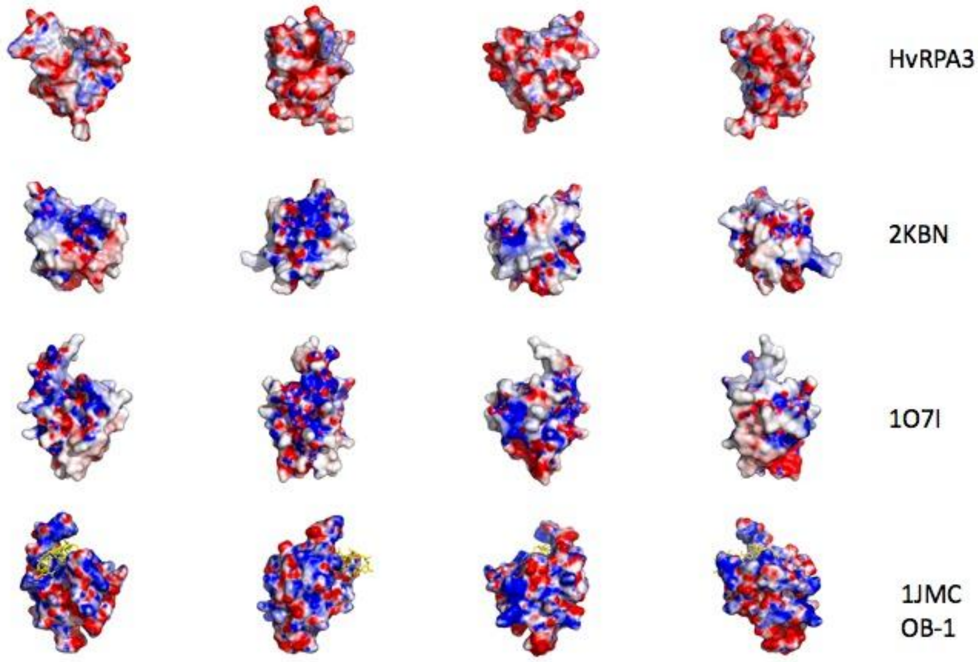

Supplementary Figure 3. Electrostatic surface potential of the HvRPA3 OB-fold model compared to related structures scaled at -10 k_BT/e (red) to +10 k_BT/e (blue). Human RPA70 shows a single OB-fold for clarity, with a 4mer stretch of oligonucleotide in stick representation in yellow. Produced using APBS and PyMol.
